# Endostatin expression in a pancreatic cell line is modulated by a TNF*α*-dependent elastase

**DOI:** 10.1038/sj.bjc.6602835

**Published:** 2005-10-18

**Authors:** R D Brammer, S R Bramhall, M C Eggo

**Affiliations:** 1 Division of Medical Sciences, University of Birmingham, Birmingham B15 2TT, UK; 2 Department of Surgery, Queen Elizabeth Hospital, Birmingham B15 2TH, UK

**Keywords:** endostatin, elastase, TNF*α*, SUIT-2, pancreas

## Abstract

Endostatin, an inhibitor of angiogenesis, is a 20 kDa fragment of the basement membrane protein, collagen XVIII. The formation of endostatin relies upon the action of proteases on collagen XVIII. TNF*α*, produced by activated macrophages, is a multifunctional proinflammatory cytokine with known effects on endothelial function. We postulated that TNF*α* may modulate the activities of proteases and thus regulate endostatin formation in pancreatic cells. Collagen XVIII/endostatin mRNA was expressed in one pancreatic cell line, SUIT-2, but not in BxPc-3. The 20 kDa endostatin was found in the cell-conditioned medium of SUIT-2 cells. Precursor forms only were found in the cells. Exogenous endostatin was degraded by cellular lysates of SUIT-2 cells. Elastase activity was found in cell extracts but not the cell-conditioned media of SUIT-2 cells. Incubation of SUIT-2 cells with TNF*α* increased intracellular elastase activity and also increased secretion of endostatin into the medium. We conclude that endostatin is released by SUIT-2 cells and that increases in intracellular elastase, induced by TNF*α*, are correlated with increased secretion. Endostatin is however susceptible to degradation by intracellular proteases and if tissue injury accompanies inflammation, endostatin may be degraded, allowing angiogenesis to occur.

Endostatin is a dose-dependent, specific inhibitor of endothelial cell growth (O’Reilly *et al*, 1997). Recent *in vitro* studies using DNA and antibody array have identified its antiangiogenic effects on microvascular endothelial cells (Abdollahi *et al*, 2004). Systemic administration of recombinant endostatin is capable of suppressing the growth of metastases with no evidence of either toxicity in phase I clinical trials or drug resistance (Folkman, 2004). Further clinical trials using endostatin as an anticancer agent, are ongoing (Davis *et al*, 2004). Endostatin is formed by the proteolytic degradation of its parent molecule, collagen XVIII (Ferreras *et al*, 2000). Since collagen XVIII is a component of the vascular basement membrane, endostatin has been found in a large number of tissues. The detection of a broad range of endostatin-like fragments in tissue and serum with sizes ranging from 20 to 38 kDa indicates that collagen XVIII is sensitive to proteolytic degradation at a number of sites. This suggests that a number of proteolytic pathways may exist for the generation and degradation of endostatin.

The *C* terminus of collagen XVIII contains the NC1 domain (38 kDa) from which 20 kDa endostatin is derived. Based on structural data, Sasaki *et al* (2000) proposed that the NC1 segment of collagen XVIII consisted of three different segments: an association domain responsible for noncovalent trimerisation, a hinge region susceptible to proteolytic degradation and the compact endostatin domain. *In vitro* studies identified secreted cathepsin L as an enzyme capable of generating endostatin, whereas the matrix metalloproteases produce larger fragments in an alternate pathway (Felbor *et al*, 2000). *In vivo*, proteolytic processing of collagen XVIII can generate both the NC1 trimer and the 20 kDa endostatin monomer (Sasaki *et al*, 1998; Wen *et al*, 1999) since both fragments are found in tissues and serum (Standker *et al*, 1997; John *et al*, 1999). *In vitro*, the NC1 fragment is more sensitive to proteolytic degradation than 20 kDa endostatin (Ferreras *et al*, 2000).

We have previously identified endostatin in human pancreatic cancer tissue and showed that it was susceptible to degradation by elastase (Brammer *et al*, 2005). We sought to identify a pancreatic cell line capable of endostatin production in order to examine the regulation of endostatin production. We hypothesised that endostatin production could be modulated by protease activity under the influence of TNF*α*, a cytokine with multifunctional effects in states of inflammation and malignancy.

There is much evidence to link proinflammatory cytokines with malignancy in the pancreas as in other tissues (Farrow and Evers, 2002; Jura *et al*, 2005, Mantovani, 2005). Early studies showed that TNF*α* produced by activated macrophages induced angiogenesis (Leibovich *et al*, 1987), which would support carcinogenesis. However, pancreatic ductal carcinoma is not a particularly vascular malignancy, exhibiting instead a heterogeneous vascularity with enhanced foci of endothelial proliferation (Korc, 2003). This suggests that local factors control the angiogenesis. Furthermore, not all cases of pancreatitis lead to malignancy also suggesting that other factors are important. Pancreatic cancer cell lines are known to produce many proteases and while some of these will aid invasion of the metastatic cells, others may promote endostatin production from collagen XVIII and thus limit tumour spread. Our studies aim to understand how endostatin production is regulated.

## MATERIALS AND METHODS

### Cell culture

SUIT-2 is a human pancreatic tumour cell line derived from a liver metastasis and is known to display a highly metastatic phenotype with spontaneous metastasis to lung and regional lymph nodes from subcutaneous nude mouse xenografts (Taniguchi *et al*, 1992). BxPc-3 were derived from an adenocarcinoma of the body of the pancreas and are moderately differentiated (Tan *et al*, 1986). Cells were cultured in Rivers Park Memorial Institute (RPMI) 1640 media supplemented with 10% foetal bovine serum and grown in an incubator at 37°C, in an atmosphere of 5% CO_2_ and 95% humidified air. Cell culture medium was supplemented with penicillin (10^5^ IU l^−1^) and streptomycin (100 mg l^−1^). Cells were tested and were negative for mycoplasma.

### Reverse transcriptase–polymerase chain reaction (RT–PCR)

Reverse transcriptase–polymerase chain reaction was used to determine whether cells expressed mRNA for collagen XVIII-endostatin. Total RNA was isolated from SUIT-2 cells, BxPc-3 cells and two samples of normal pancreatic tissue using Trizol (Gibco-BRL, Paisley, UK) as described by the manufacturer. The RNA was dissolved in RNAase-free, diethyl pyrocarbonate-treated water and stored at −70°C until use.

Primers were designed using Primer 3 software (http://www.genome.wi.mit.edu/cgi-bin/primer/primer3-www.cgi) from the sequences for endostatin listed on GeneBank. The primers were

ENDO SENSE: CTCAATGCAGAGCACGATGT
ENDO ANTISENSE: TGTTCTCAGGCTCTGAGGGT

The RT–PCR reaction was performed using Ready-To-Go RT–PCR beads (Amersham Biosciences, UK) according to the manufacturer's instructions. After the RT reaction for 30 min at 42°C, the samples were heated to 94°C for 5 min, then through 35 cycles of 95°C for 1 min, 55°C 1 min and 72°C for 2 min. The expected product size was 269 bp.

The samples were run at 50 V on 1% agarose gels for 2–3 h in Tris-borate-EDTA buffer and ethidium bromide-labelled bands visualised on the gel under an ultraviolet lamp. Known molecular weight standards were run concomitantly to allow sizing of the products.

### Western blotting

Western immunoblotting, which separates immunoreactive fragments of collagen XVIII by molecular weight was employed to detect endostatin (20 kDa). SUIT-2 cells were cultured until subconfluent and then cultured for 2 or 3 days in serum-free media. Secreted proteins were collected by precipitation from three volumes of ethanol and dissolved in reducing sample buffer (2% sodium dodecyl sulphate (SDS), 62.5 mM Tris-HCl, 10% glycerol, 10% 2-mercaptoethanol, pH 6.8) for SDS-polyacrylamide gel electrophoresis (SDS–PAGE). The cell layer proteins were also analysed by SDS–PAGE to allow comparison between secreted and nonsecreted endostatin-related fragments. Detection was as described previously (Brammer *et al*, 2005) using enhanced chemiluminescence with rabbit polyclonal antisera to human endostatin (Chemicon, Hampshire, UK) diluted 1 : 100. The specificity of the antisera was shown by preabsorption of the antibody with an excess of recombinant endostatin. Preabsorbed antisera did not bind to any proteins on Western blots of pancreatic tissue, whereas the nontreated antisera reacted with discrete proteins of various sizes including that of full-length collagen XVIII.

### Subcellular fractionation of SUIT-2 cells

SUIT-2 cells were scraped from dishes and suspended in 1 ml of HBSS. The cells were sonicated in an ice bath to avoid heating during disruption. The suspension was centrifuged at 3000 **g** for 30 min and the supernatant was diluted to a protein concentration of 1 mg ml^−1^ with HBSS pH 7.2. Five nanograms of recombinant endostatin was incubated with 150 *μ*l of SUIT-2 cell lysate (1 mg ml^−1^ protein) and incubation at 37°C was terminated at various time points by freezing at −20°C. A volume of 50 *μ*l of 5 × reducing sample buffer was added to the samples which were heated at 100°C for 10 min before separation by SDS–PAGE.

### Elastase activity in SUIT-2 cells

Activity of elastase in the pancreatic cell line was evaluated using a colorimetric assay with *N*-methoxysuccinyl-Ala–Ala–Pro–Val P-nitroanilide as a chromogenic substrate as described by Bieth and Wermuth (1973). A total of 10 million SUIT-2 cells were suspended in 0.5 ml of 20 mM HEPES buffer, 0.25 M sucrose pH 7.2, and sonicated in an ice bath to avoid heating during cell disruption. Five 30 s pulses were used. The suspension was centrifuged at 1000 **g** for 10 min and the supernatant was centrifuged again at 2000 **g** for 30 min. The supernatant was diluted to a protein concentration of 1 mg ml^−1^ with 20 mM HEPES, 0.25 M sucrose, pH 7.2.

The enzyme kinetics of the pancreatic cellular extract were evaluated and the sensitivity of the assay was verified using purified elastase (Sigma, UK). Incubation was performed at room temperature and the plate was read at intervals between 5 and 30 min up to a period of 24 h. The results were analysed by plotting a Lineweaver–Burk curve to determine the Km and Vmax.

### Effect of TNF*α* on endostatin production and degradation

The effect of the cytokine TNF*α* was investigated to determine whether endostatin production from collagen XVIII could be modulated by cytokines. Secretion of endostatin into the media was examined. The effect of cell extracts on endostatin degradation was determined as described in the previous section.

SUIT-2 cells were cultured for 48 h in serum-free RPMI media in 9.6 cm^2^ culture plates in the presence of TNF*α*. After removal from the cells, 1 ml of the cell-conditioned medium was incubated with 5 ng of recombinant endostatin at 37°C for 4 h to determine whether degradation of the exogenous endostatin occurred as a result of proteases released into the media. The secreted proteins were concentrated from the cell-conditioned culture medium by ethanol precipitation and analysed by SDS–PAGE and immunoblotting.

## RESULTS

### RT–PCR for collagen XVIII/endostatin

**Figure 1:**
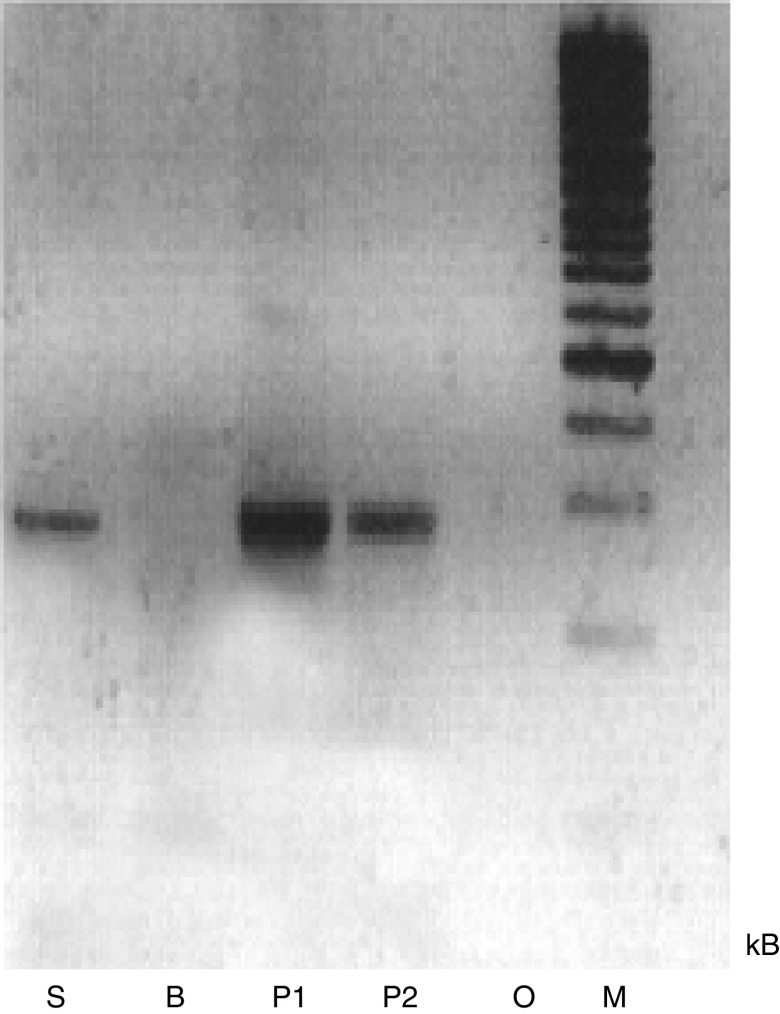
RT–PCR showing collagen XVIII-endostatin mRNA expression in pancreatic tissue and pancreatic cancer cell lines. S=Suit-2, B=BxPc3, P1&P2=normal pancreas, O=water control.

A single band of approximately 269 bp corresponding to the expected product size was seen with two samples of normal pancreatic tissue and the pancreatic cell line, SUIT-2 as shown in Figure 1. BxPc-3 cells did not express mRNA for collagen XVIII and were not used further.

### Collagen XVIII-endostatin expression by the SUIT-2 cell line

**Figure 2:**
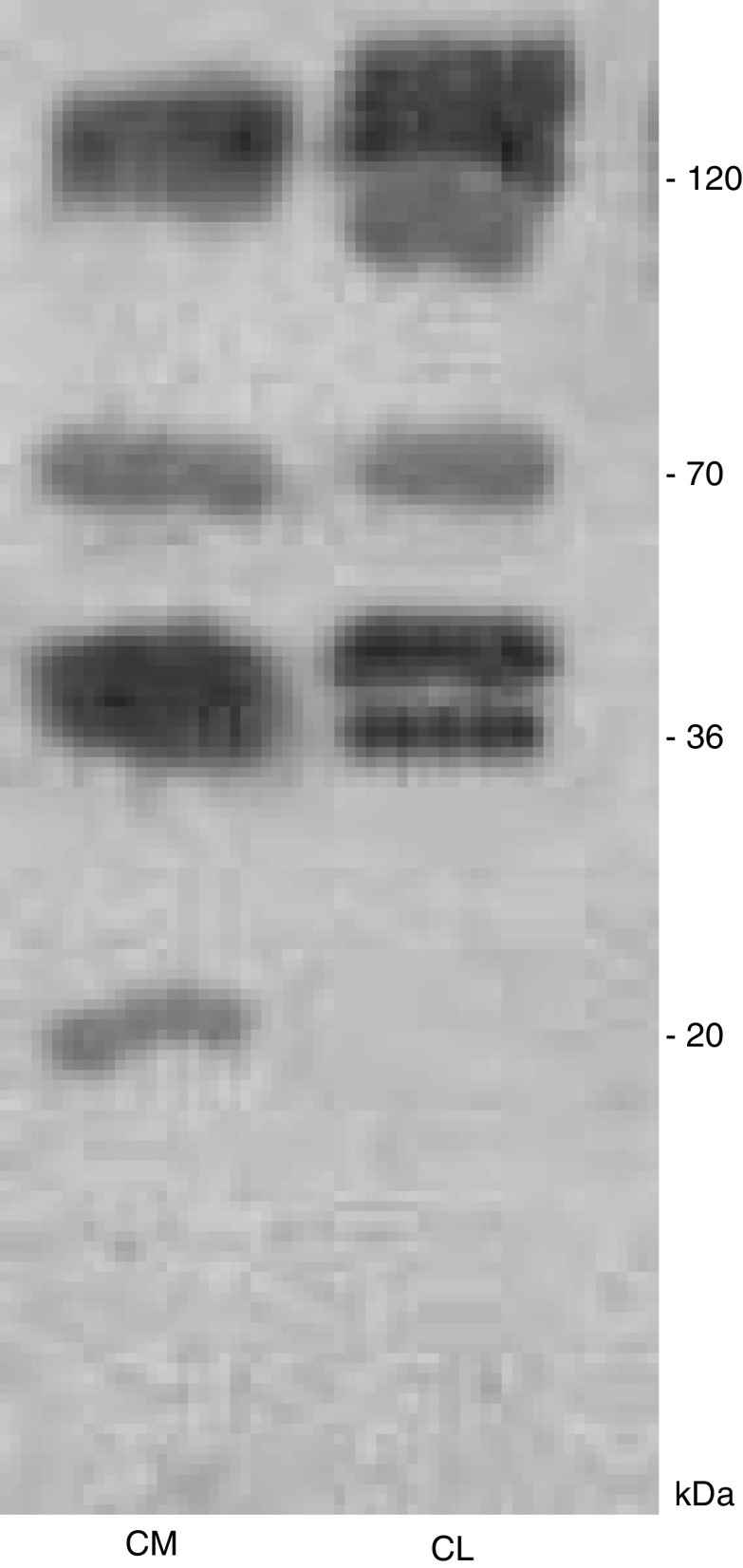
Western immunoblotting of SUIT-2 cell layer and cell-conditioned media probed for endostatin (CM=cell media; CL=cell layer).

Western blotting, shown in Figure 2, demonstrated that not only collagen XVIII but also 20 kDa endostatin are produced by SUIT-2 cells. Endostatin is secreted into the cultured cell media but not found in the cell layer. The higher molecular weight forms of endostatin-related proteins were found in both the cell layer and the cell-conditioned media and were of similar size, the predominant products being 36–38 kDa which is likely to be the NC1 domain, and 70 kDa.

### Endostatin degradation by SUIT-2 cellular lysate

**Figure 3:**
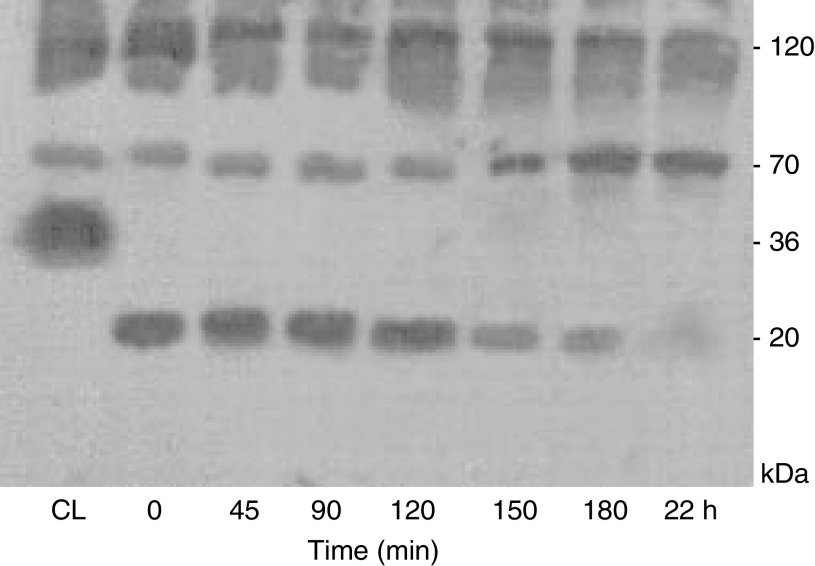
Western immunoblotting showing degradation of recombinant endostatin with time by SUIT-2 cellular lysates probed for endostatin. (CL=cell layer without homogenisation).

The time course of degradation of exogenous endostatin by lysates of SUIT-2 cells is shown in Figure 3. Visible evidence of endostatin degradation was seen by 150 min. By 22 h none remained. The band at 70 kDa became more prominent with incubation and this corresponded to a reduction in the 120 kDa band. Interestingly, the 36 kDa endostatin-related fragment was very susceptible to degradation. The difference between the CL and the *t*=0 is that the CL sample was immediately dissolved in reducing sample buffer whereas the *t*=0 extract had been sonicated and centrifuged. There is complete loss of the 36 kDa band during the homogenisation period.

### Elastase activity in SUIT-2 cells

**Figure 4:**
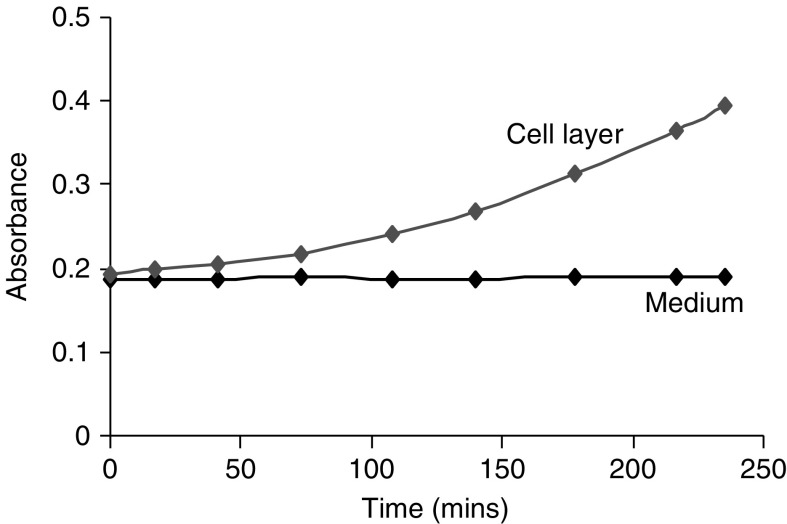
Rate of hydrolysis of the elastase substrate by SUIT-2 cell extract and serum-free cell-conditioned culture media.

Elastase activity, measured by the colorimetric method, in the cell extracts and in 48 h collection of serum-free cell conditioned medium is shown in Figure 4. There was detectable elastase in the cell layer yet there was none in the 48 h cell-conditioned medium.

**Figure 5:**
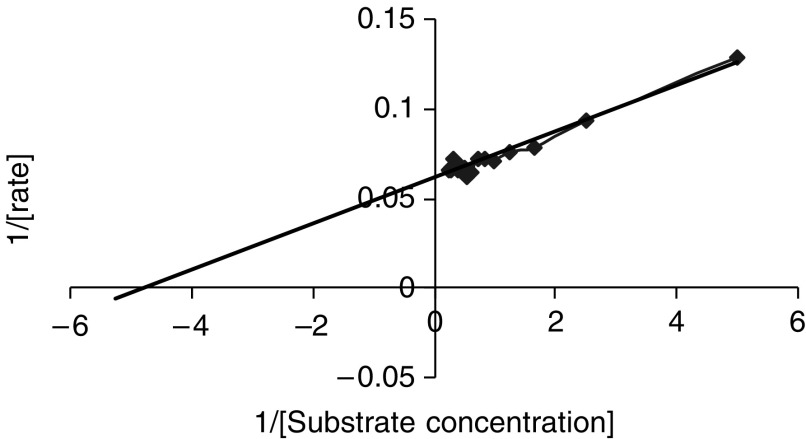
A Lineweaver–Burk plot for assay of intracellular SUIT-2 elastase activity.

From the Lineweaver–Burk curve shown in Figure 5, we were able to calculate the Vmax and Km for the reaction as 16 mM s^−1^ and 0.2 mM, respectively. This compares with the Km of 1.1 mM for an elastase we identified in human pancreas (Brammer *et al*, 2005) and human leukocyte elastase where the Km is reported to be 0.14 mM (Castillo *et al*, 1979).

### Effect of TNF*α* on endostatin production from SUIT-2 cells

**Figure 6:**
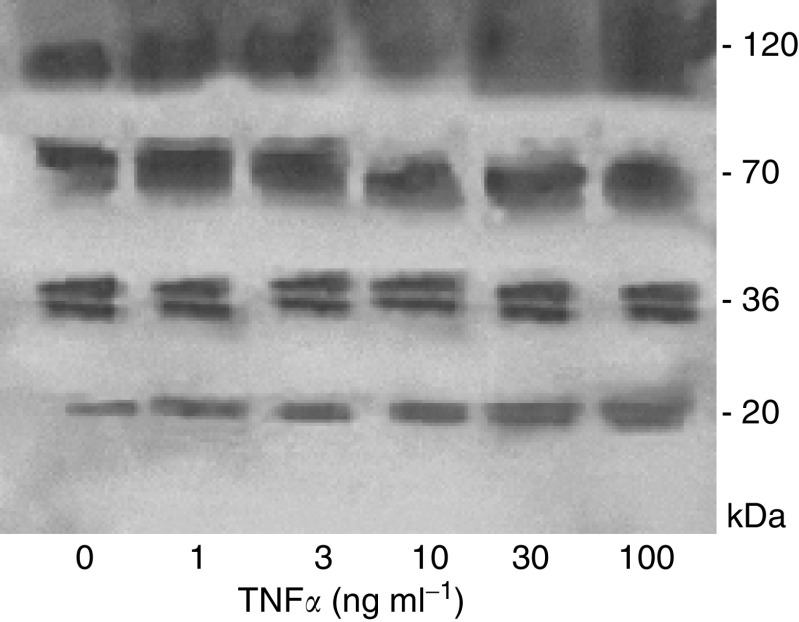
Western immunoblotting showing dose-dependent effects of TNF*α* on secretion of endostatin and endostatin-related proteins into culture media by SUIT-2 cells treated with varying doses of TNF*α* for 48 h.

Figure 6 shows the effect of incubation of SUIT-2 cells with TNF*α* for 48 h on endostatin secretion. The 20 kDa endostatin in the cell-conditioned medium was increased in a dose-dependent manner following 48 h treatment with TNF*α*. The intensity of the other endostatin-related fragments was unchanged.

### Effect of TNF*α* on endostatin degradation and elastase activity

**Figure 7:**
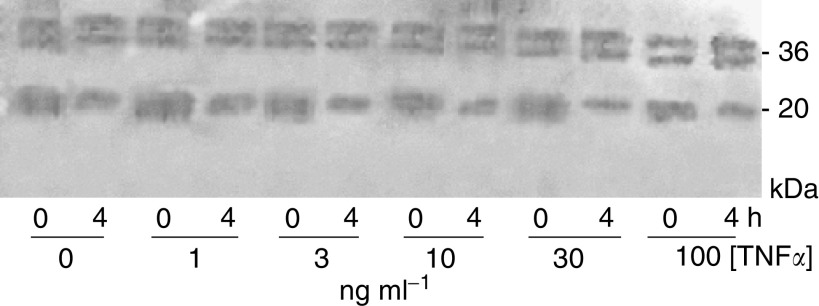
Western immunoblotting showing effect of incubating exogenous endostatin with cell-conditioned media from cells treated with varying doses of TNF for 48 h. 0 and 4 h incubation times are shown.

Figure 7 shows the effects of incubating exogenous endostatin with the cell-conditioned medium from the experiment described in Figure 6. Although there was appreciable degradation of exogenous endostatin following the 4 h incubation, there were no convincing changes with increasing dose of TNF*α*.

### Effect of TNF*α* on elastase activity

**Figure 8:**
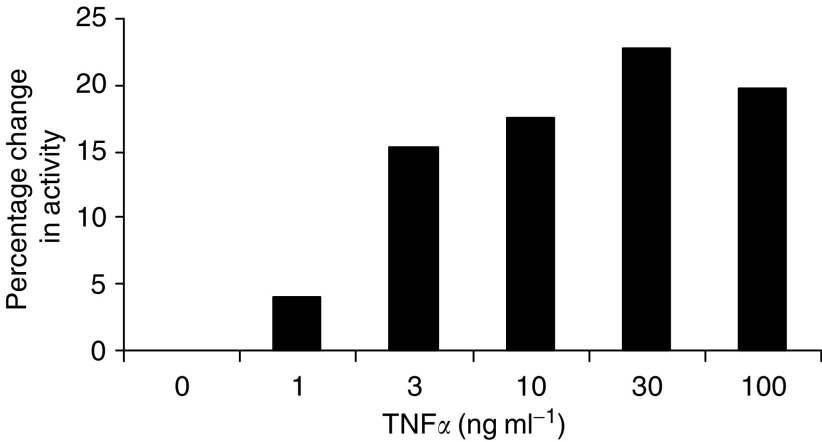
Dose-dependent effect of TNF*α* upon intracellular elastase activity in the SUIT-2 cell layer shown as percentage change in activity.

Figure 8 shows the effect of TNF*α* on intracellular elastase activity, measured using the colorimetric assay. The results are shown as percentage change from control. Incubation with TNF*α* increased intracellular elastase activity in a dose-dependent manner in the SUIT-2 cell layer.

## DISCUSSION

We have shown that the SUIT-2 cells, but not BxPc-3 cells, express the mRNA for collagen XVIII. Western immunoblotting confirmed that these cells can produce mature 20 kDa endostatin from collagen XVIII and therefore possess the necessary proteolytic enzymes required for synthesis. Mature endostatin was found in the cell-conditioned media where higher molecular weight forms of endostatin-related proteins were also found. This suggests that collagen XVIII, which is normally considered as an integral membrane protein, can be readily released into medium. Whether this is due to enzymatic action is not clear. The 20 kDa form of endostatin was not present in the cell layer, however collagen XVIII and several immunoreactive precursors of endostatin were present. The absence of the 20 kDa endostatin within the cells implies that either intracellular endostatin is degraded or that endostatin is processed at the cell membrane from the larger fragments of collagen XVIII and secreted.

We found that extracts of SUIT-2 cells were capable of degrading endostatin and endostatin-related fragments of collagen XVIII. The 36 kDa endostatin-related fragment was particularly susceptible to proteolysis, once the subcellular organisation of cells was destroyed. The central protease-sensitive region of the NC1 fragment may undergo proteolysis to a variable extent by a number of proteases such as cathepsins D and L which are lysosomal enzymes, matrix metalloproteases and elastase. Cathepsins are produced by both endothelial cells and tumour cells and these proteases are activated by the acidic pH that is found in the surroundings of tumours. Although they may have a role in endostatin generation (Felbor *et al*, 2000), they may also be important in endostatin degradation. We previously implicated elastase in the degradation of endostatin in extracts of normal pancreas (Brammer *et al*, 2005). The release of intracellular proteases that degrade endostatin following tissue injury, may be a means to reduce local endostatin levels. This would allow angiogenesis to occur and thus initiate healing but may also aid in tumour establishment.

TNF*α* increased the secretion of endostatin in a dose-dependent manner but did not alter the release of intermediate endostatin-related fragments of collagen XVIII. TNF*α* treatment may therefore act by increasing the activity of an intracellular protease producing endostatin from collagen XVIII. TNF*α* increased intracellular elastase activity which was correlated with increased levels of secreted endostatin. We did not find secreted elastase activity but we did show that exogenous endostatin was lost from the medium. Either there is secretion of proteases, which presumably are not elastases, which degrade endostatin or endostatin is taken up and degraded by the intracellular proteases.

We characterised the intracellular elastase expressed in SUIT-2 cells. We found that the Km for this enzyme differed from that of the human pancreatic elastase we had previously characterised and from human leucocyte elastase. Yamaguchi *et al* (2000) also described a novel elastase in these cells, distinct from human leucocyte elastase, which had similar activity to neutrophil elastase but was immunologically different. Furthermore, this novel elastase was not inhibited by neutrophil elastase and pancreatic elastase inhibitors.

We conclude that SUIT-2 cells can synthesise and secrete endostatin and that this synthesis can be increased by TNF*α*. Endostatin would oppose the known effects of TNF*α* to increase angiogenesis by increasing the secretion of vascular endothelial growth factors (VEGF) (Farrow and Evers, 2002; Korc, 2003). Clinically, increased production of endostatin from normal pancreatic cells due to TNF*α* production from macrophages would limit healing of the inflamed pancreas. Where the balance lies in these antagonistic effects is likely to vary and may explain the heterogeneous angiogenesis seen in pancreatic disease.

If endostatin production were increased, and angiogenesis therefore limited, this would result in hypoxia. Hypoxia increases the expression of VEGF by the cells which stimulates angiogenesis (Farrow and Evers, 2002; Korc, 2003; Jura *et al*, 2005). Hypoxia however induces many other changes in the proteome which promote cell survival, proliferation and spread. Cells adapt to changes in nutrient availability by changes the expression of glucose transporters and glycolytic enzymes (Vaupel and Harrison, 2004). These changes may promote the selection of tumour cells that are able to survive adverse conditions. This hypothesis could explain clonal selection of malignant cells from inflamed pancreatic tissue that are more aggressive and capable of metastasising.
